# Recycling of Sewage Sludge: Synthesis and Application of Sludge-Based Activated Carbon in the Efficient Removal of Cadmium (II) and Lead (II) from Wastewater

**DOI:** 10.3390/ijms25189866

**Published:** 2024-09-12

**Authors:** Salha M. Aljubiri, Ayman A. O. Younes, Eid H. Alosaimi, Mahmoud M. Abdel Daiem, Enas T. Abdel-Salam, Walaa H. El-Shwiniy

**Affiliations:** 1 Department of Chemistry, College of Science, University of Bisha, Bisha 61922, Saudi Arabia; sragh@ub.edu.sa (S.M.A.); aayounes@ub.edu.sa (A.A.O.Y.); ealosaimi@ub.edu.sa (E.H.A.); enastaha@ub.edu.sa (E.T.A.-S.); 2 Environmental Engineering Department, Faculty of Engineering, Zagazig University, Zagazig 44519, Egypt; mmabdeldaiem@eng.zu.edu.eg; 3 Civil Engineering Department, College of Engineering, Shaqra University, Al-Duwadmi 11911, Saudi Arabia; 4 Department of Chemistry, Faculty of Science, Suez Canal University, Ismailia 41522, Egypt; 5 Chemistry Department, Faculty of Science, Zagazig University, Zagazig 44519, Egypt

**Keywords:** adsorption, low-cost adsorbents, isotherms, wastewater and heavy metals

## Abstract

The limited supply of drinking water has aroused people’s curiosity in recent decades. Adsorption is a popular method for removing hazardous substances from wastewater, especially heavy metals, as it is cheap, highly efficient, and easy to use. In this work, a new sludge-based activated carbon adsorbent (thickened samples SBAC1 and un-thickened samples SBAC2) was developed to remove hazardous metals such as cadmium (Cd^+2^) and lead (Pb^+2^) from an aqueous solution. The chemical structure and surface morphology of the produced SBAC1 and SBAC2 were investigated using a range of analytical tools such as CHNS, BET, FT-IR, XRD, XRF, SEM, TEM, N_2_ adsorption/desorption isothermal, and zeta potential. BET surface areas were examined and SBAC2 was found to have a larger BET surface area (498.386 m^2^/g) than SBAC1 (336.339 m^2^/g). While the average pore size was 10–100 nm for SBAC1 and 45–50 nm for SBAC2. SBAC1 and SBAC2 eliminated approximately 99.99% of Cd^+2^ and Pb^+2^ out the water under all conditions tested. The results of the adsorption of Cd^+2^ and Pb^+2^ were in good agreement with the pseudo-second-order equation (R^2^ = 1.00). Under the experimental conditions, the Cd^+2^ and Pb^+2^ adsorption equilibrium data were effectively linked to the Langmuir and Freundlich equations for SBAC1 and SBAC2, respectively. The regeneration showed a high recyclability for the fabricated SBAC1 and SBAC2 during five consecutive reuse cycles. As a result, the produced SBAC1 and SBAC2 are attractive adsorbents for the elimination of heavy metals from various environmental and industrial wastewater samples.

## 1. Introduction

The worldwide demand for including environmental sustainability into all aspects of the practice of management is developing [1,2]. Sewage sludge handling and management is one of the fast-rising concerns that needs long-term attention, as it makes up about half of the entire treatment cost [2,3]. In reality, sewage sludge is a provenance of toxins in the environment, such as microorganisms [2,4,5]. Reusing sewage sludge as fertilizer in agriculture is one of the most environmentally friendly methods of dealing with it [6]. However, because sewage sludge includes organic pollutants that might be put into agricultural soil, this strategy raises the potential health risk [5]. Although landfilling sludge with methane recovery is desirable, it is seldom performed [7]. Various substances were utilized as precursors to make activated carbon using various activation processes. Every substance is distinct from the others in terms of its chemical makeup and ability to remove heavy metals [7]. Sewage-sludge-based activated carbon (SBAC) is mostly employed in the elimination of toxic metals from wastewater as well as the removing and purification of gases [8,9]. The persistent high demand for activated carbon and the high cost of manufacturing are a result of significant environmental issues [10]. Therefore, a lot of research has been carried out to investigate the utilization of various wastes as an inexpensive precursor to manufacturing activated carbon [11]. Sewage sludge is an undesirable byproduct of cleaning up wastewater that consists of organic and inorganic pollutants [2]. The volume of sewage sludge is increasing due to rapid population development and urbanization [2,12]. As a result, the management and disposal of sewage sludge is seen as a serious challenge, necessitating the development of alternate disposal and management approaches [13]. Sewage sludge is a carbonaceous substance that may be utilized to make activated carbon at a cheap price to remove a variety of contaminants from water and air [14]. Sludge is demonstrated to generate good-quality carbons for the adsorption of harmful substances in water, such as heavy metals and pigments. Producing SBAC is estimated to be an economical option for both the disposal of waste and the manufacture of cheap adsorbents [14]. Chemical pollutants enter the environment from a variety of sources, including manufacturing, mineral extraction, and agricultural operations. Heavy metals are of particular concern due to their hazardous effects, persistence, and lack of biodegradable. Detecting heavy metal contamination in the environment is so critical [15]. Cadmium is extensively employed in nickel–cadmium (Ni–Cd) batteries and as a coating for iron and steel, alloys, mining, metal finishing, textile operations refining plants, solar cells, plastic stabilizers, and pigments. Cadmium may gradually accumulate in organisms, mostly via the food web. Exposure to cadmium in water may impact organs, including the lungs, kidneys, liver, immunological, cardiovascular, and reproduction [16]. The major sources of lead change by region including lead reuse and recycling, the industrial usage of lead in paintings and as an ingredient in gasoline, and the lead pipe used in water distribution systems [16,17]. The World Health Organization (WHO) has set a maximum detection limit of 0.003 mg L^−1^ for cadmium and 0.01 mg L^−1^ for lead in drinking water, while the USEPA has set a standard of 0.002 mg L^−1^ and 0.005 mg L^−1^ due to the hazardous consequences of cadmium and lead in the environment, respectively [18,19]. There are several techniques for removing heavy metal ions from wastewater, such as adsorption, separate membranes, exchange of ions, electroplating, and precipitation. The most widely used of these methods for eliminating pollutants is adsorption, which makes use of a variety of absorbent types, including synthetic, natural, organic, inorganic, activated, and modified materials [20]. The aim of this study is, therefore, to provide information on the use of cheap activated carbon adsorbents with excellent adsorption potency against two harmful heavy metals (cadmium and lead ions) by employing the sewage sludge as carbon precursors. The physical and chemical properties of the sludge-based activated carbon (SBAC) will be investigated to gain insight into the material, which will then be used to correlate with ion absorption in the aqueous phase. The starting metal concentration, adsorbent dose, pH, contact duration, and temperature were all considered while determining the efficiency of the generated adsorbents. In addition, the kinetic and equilibrium sorption of Cd^+2^ and Pb^+2^ ions and the modeling of their transfer to the surface of sludge-based activated carbon will be investigated. The results demonstrate that the affordable SBAC adsorbents developed might be used to effectively remove heavy metals for environmental cleanup.

## 2. Results and Discussion

### 2.1. The Ultimate Analysis of Activated Carbon

The final analytical findings for the sewage sludge samples are displayed in Table 1. The differences in the content of C, H, O, and N and the H/C, O/C, N/C ratio demonstrate that the composition of the organic materials of the sewage sludge was altered by the two-hour paralysis at 573 K (300 °C) and the subsequent one-hour paralysis at 1173 K (900 °C). Compared to the raw sewage sludge (i.e., raw sewage sludge), the H/C, N/C and O/C ratios decreased after paralyzing. The H/C ratio decreases 0.14 (raw SS) to 0.05 (SBAC1) and 0.06 (SBAC2). The phenomenon of fluctuations in the H/C, N/C, and O/C ratios demonstrates that, compared to the raw SS samples, the paralyzed sewage sludge samples have more aromatic groups. This might be the result of the digestion of light organic compounds during the paralysis method [21]. One peculiarity is that the SBAC2 sample has a higher H/C ratio than the other SBAC1 samples. It, therefore, suggests that the paralyzed non-thickened sample has the best results when lengthy chains (containing CH_2_ groups) and high aliphatic carbon content are present [22,23]. Producing gaseous alkanes or light aromatic hydrocarbons has advantages from the higher aliphatic carbon content. The degree of organic material polymerization in the sewage sludge can be expressed using the N/C ratio. Less functional groups containing nitrogen can be found in higher polymerized organic material [23,24]. The higher degree of polymerization of the paralyzed sewage sludge thus implies a greater potential for dewatering. As stated in Table 1, the O/C ratios of SBAC1 and SBAC2 are considerably less than those of raw sewage sludge, indicating that some of the oxygen may be transferred from oxygen-containing functional groups or oxidized during paralysis.

### 2.2. X-ray Fluorescence (XRF) Analysis

The ash was usually produced by metal ions in the wastewater. SBAC1 and SBAC2 were collected during the final treatment of the raw sewage sludge. During the raw sewage sludge’s final treatment, SBAC1 and SBAC2 were collected for analysis. The water was filtered during this process by the different metal ions being adsorbed on the surfaces of SBAC1 and SBAC2. Both thermal treatment and chemical activation did not cause the deposited metal ions to evaporate; instead, they stayed on the carbon surface and in the pores. Ash was created from the metal ions, and this covered the activated carbon surface and sealed the pores [25]. They inhibited the use of active carbon and decreased the surface area. XRF was used to examine the adsorbed metal ions (Table 2). The Si, Ca, and Fe ions were mainly bound in the raw sewage sludge (RSS). In SBAC1 and SBAC2, the metal composition was not significantly different. The Fe, Si, Cu, Zn, Mn, and Ni contents overall were essentially greater in SBAC1 and SBAC2 than they were in the raw sewage sludge; this can result from the fact that, during pyrolysis, heavy metals lose less weight than organic substances, causing heavy metals to build up in the SBAC1 and SBAC2 matrix [26]. In general, the heavy metal concentrations in SBAC1 and SBAC2 and the sludge were in the following order: Ca > Si > Fe > P > K > Al.

### 2.3. FT-IR Analysis

**Figure 1:**
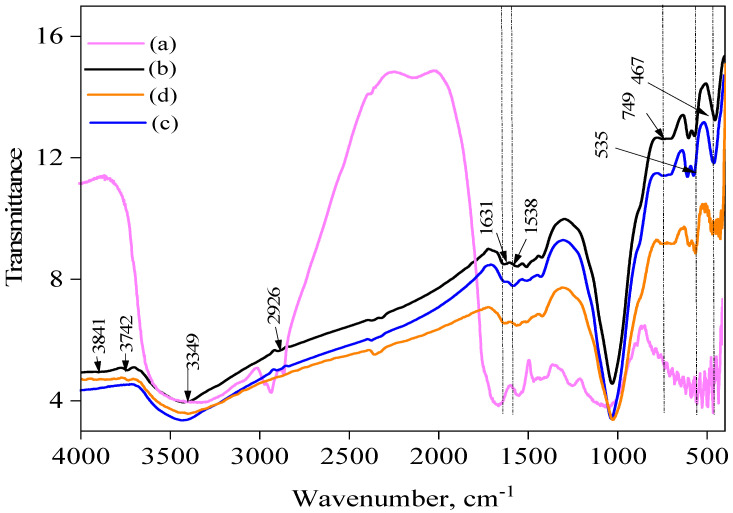
FTIR spectra of (a) thickened sewage sludge, (b) SBAC1, (c) SBAC1 after absorption Cd^+2^, and (d) SBAC1 after absorption Pb^+2^.

**Figure 2:**
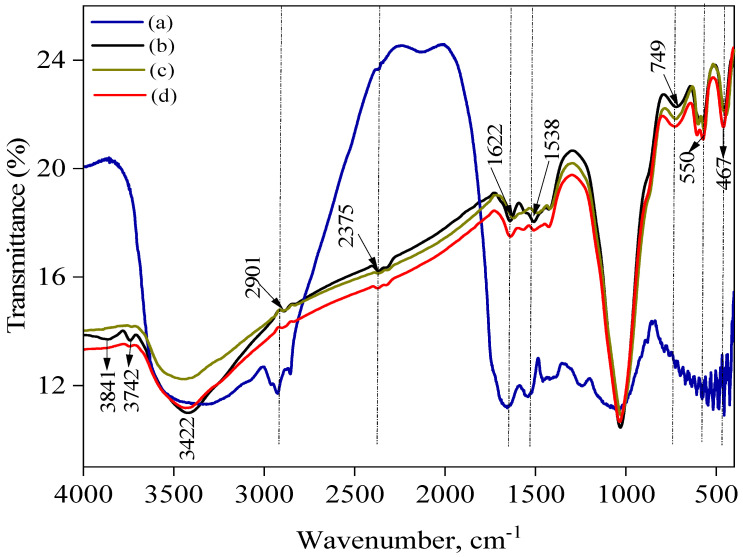
FT-IR spectra of (a) unthickened sewage sludge, (b) SBAC2, (c) SBAC2 after absorption Cd^+2^, and (d) SBAC2 after absorption Pb^+2^.

The FT-IR spectra of the raw sewage sludge are displayed in Figure 1 and Figure 2 and show wavenumbers in the range of 400 to 4000 cm^−1^. When looking from left to right, the peak at 3688–3619 cm^−1^ represents the lattice stretching of OH–kaolinite and gibbsite [27,28], 2988–2901 cm^−1^ the -C–H group’s vibration [29], and 1631 cm^−1^ and 1538 cm^−1^ the functional groups of nitrogen and sulfur, respectively [29]. The shoulder peaks’ rise at 1050–1090 cm^−1^ was connected to the Si–C or Si–O–Si bands, C-O-C vibrations [30], and C-O-C vibrations [30], and, ultimately, silica or calcium carbonate stretching was identified as the cause of the peaks at 749 cm^−1^, 535 cm^−1^, and 467 cm^−1^ below 1000 cm^−1^ [27,31]. Functional groups including carboxyls, phenols, aldehydes, ketones, quinones, hydroquinones, and anhydrides have been confirmed to be present on activated carbon surfaces [32]. These functional groups define which carbon compounds are acid–base. The oxygen-containing groups are responsible for the acidic and basic properties [32]. The absorption band at 3841 and 3736 cm^−1^ was assigned to the (O-H) vibrations of the hydroxyl groups. Adsorbed water may be the cause of the location of the OH groups involved in hydrogen bonding, which are typically found in the range of 3394–3841 cm^−1^ for alcohols and phenols [33]. While the C=C stretching vibration of aromatic rings is indicated at 1622 cm^−1^, the C=O stretching of ketones, aldehydes, or carboxyl groups is marked at 2375 cm^−1^. A significant absorption band at 1053 cm^−1^ is seen in SBAC1 and SBAC2, and it is linked to either the Si–O–Si or Si–O–C structures. This band is related to the silicon content in the sewage sludge. The band at 617 cm^−1^ is broad and represents the C–O–H twist. Examining the spectra in more detail shows that, at a high pyrolysis temperature, the baseline moves upwards from low to high wavenumbers. This is most likely due to the increased aromatic content of the charcoal during the pyrolysis process [34]. A comparison of the spectra of SBAC1 and SBAC2 before and after the sorption of Cd^+2^ and Pb^+2^ reveals differences in the position of the absorption peaks. The stretching vibration of the hydroxyl group peaks at 3742 cm^−1^ and 3642 cm^−1^ clearly disappeared after the adsorption of Cd^+2^ and Pb^+2^, indicating that chemical interactions occur between the metal ions and the hydroxyl groups on the activated carbon surface. A shift was observed in the C=O band (1697 cm^−1^ to 1587 cm^−1^).

### 2.4. Characteristics of the Porous Structure

**Figure 3:**
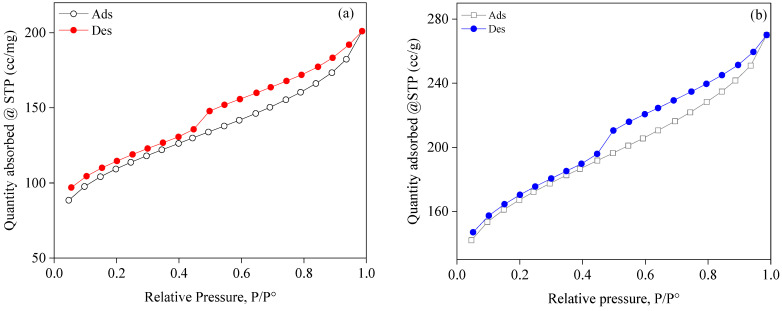
N_2_ adsorption and desorption isotherms of (**a**) SBAC1 and (**b**) SBAC2 samples.

The N_2_ adsorption and desorption isotherms for SBAC1 and SBAC2 are displayed in Figure 3. An isotherm’s lower branch displays adsorption readings, while the upper branch displays desorption observations. The Brunauer–Deming–Deming–Teller (BDDT) classification system states that activated carbons SBAC1 and SBAC2 exhibited a type I nitrogen isotherm as they showed a hysteresis loop and a rapid rise at low relative pressure, as shown in Figure 3. Since the hysteresis loop was related to the capillary condensation of mesoporous solids and the sharp increase was in the micropore filling, this indicated that they were microporous coals with a clear development of mesoporosity [23,35] According to de Boer’s classification, each of the three isotherms showed a type B hysteresis loop, indicating parallel, slit-shaped pores, as the desorption branches were steep at intermediate relative pressure and the adsorption branches were steep at saturation vapor pressure [36]. The isotherms of SBAC1 and SBAC2 showed a mean hysteresis loop that deviated somewhat from type I, indicating that micropores constituted only a small fraction of the two materials, considering that the adsorption process does not end at 1.0 P/P^0^ and that micropore filling essentially takes place at comparatively low partial pressures < 0.1 P/P^0^. Table 3 lists the structural parameters that were determined using the nitrogen isotherms. These values include the average pore diameter (V_Ave_) for BJH desorption, the BET total surface area (SBET), and the total pore volume of single-point adsorption (V_Total_) estimated at P/P^0^ = 0.98. As can be seen, SBAC2 has a larger BET surface area (498.386 m^2^/g) than SBAC1. SBAC2 had a lower V_BJH_ (cm^3^ g^−1^) than SBAC1, but a higher V_T_ (cm^3^ g^−1^) than SBAC1.

### 2.5. Zeta Potential Results

In order to gain insight into potential interactions between the SBAC1, SBAC2, and the Cd^+2^ and Pb^+2^ ions throughout the process of adsorption, the zeta potential was evaluated. The zeta potential’s mean value of SBAC1 and SBAC2 was −11.50 mV and −8.85 mV, respectively. They definitively show the generated active carbon’s negative charge. This result suggests that these activated carbons are more effective at adsorbing positively charged contaminants.

### 2.6. X-ray Diffraction

**Figure 4:**
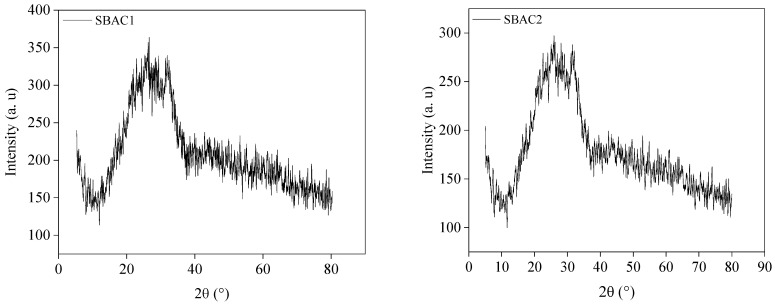
XRD spectra of produced activated carbons SBAC1 and SBAC2.

The X-ray diffraction patterns of SBAC1 and SBAC2 are shown in Figure 4 and the crystallite characteristics extracted from the XRD are summarized in Table 4. The X-ray diffraction spectrum of activated carbon formed from sludge by chemical activation shows only a few peaks, since most of the identified peaks disappear when the sludge is processed with acids, as the relevant minerals are removed by activation and rinsing with water [20]. There were two diffraction peaks SBAC1 and SBAC2 at about 2θ = 26.6° and 31°, which matched the characteristic diffraction peaks of the carbon surface (100) (JCPDS, Card No. 75-1621) [21]. The peaks showed that disordered carbon crystallites were present in both SBAC1 and SBAC2. The distance between the layers of SBAC1 and SBAC2 could be calculated using Bragg’s law and amounted to 3.35 and 2.84 nm, respectively. As a result, the SBAC2’s interfacial spacing d(001) decreased while its degree of graphitization increased. Because the activation procedure damaged the structure of the active carbon crystal and produced a thin carbon sheet that was primarily disordered, the intensity of the (100) diffraction peak of SBAC2 was noticeably diminished. Simultaneously, there was a correlation between reduced crystallite strength and increased orientation freedom of the carbon crystallites. This led to an increase in the number of pores between the crystallites and an increase in the activity of the SBAC2 sample. This resulted in a more porous structure and a greater specific surface area (498.386 m^2^ g^−1^) which improved the adsorption property of SBAC2 [22]. The d(001) values were found to be in the 16.5065 nm and 18.1714 nm range for SBAC1 and 15.0883 nm–18.0286 nm for SBAC2. These interlayer spacings are larger than the value of 0.335 nm expected for ideal graphite [35]. Such larger spacings can be related to a more disordered nature of the carbon materials herein studied which may facilitate ion penetration and, therefore, increase the adsorption properties of SBAC1 and SBAC2.

### 2.7. Microstructure of Activated Carbon

**Figure 5:**
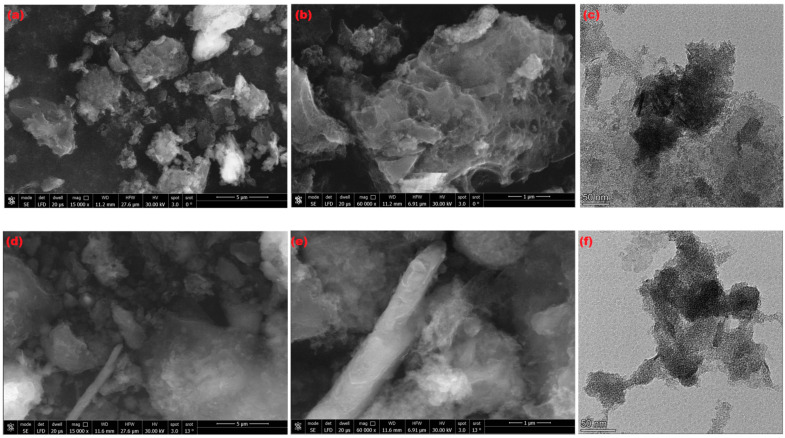
SEM micrographs of produced activated carbons SBAC1 (**a**,**b**), SBAC2 (**d**,**e**), and TEM image (**c**) SBAC1 and (**f**) SBAC2.

The morphology of the activated carbons SBAC1 and SBAC2 was investigated using scanning electron microscopy. Figure 5a,b,d,e shows microscopic images of the activated carbon. A heterogeneous distribution of particle size and shape was observed in the photomicrographs taken at low magnification. When the analytical magnification was increased, the particles appeared more porous and showed the existence of macropores and ultramacropores. This is extremely effective for the removal of impurities from water by adsorption methods. TEM was used to study the microstructure of sludge-based activated carbons SBAC1 and SBAC2 (Figure 5c–f). TEM analysis revealed that SBAC1 and SBAC2 have extensive mesoporous properties. The distribution of particles size is about 10–100 nm for SBAC1 and 45–50 nm for SBAC2, which is consistent with the results of XRD analysis. The pore structure of SBAC1 and SBAC2 is important for pollutant adsorption (Cd^+2^ and Pb^+2^ ions).

### 2.8. Adsorption Study

#### 2.8.1. Influence of the SBAC1 and SBAC2 Dosage

**Figure 6:**
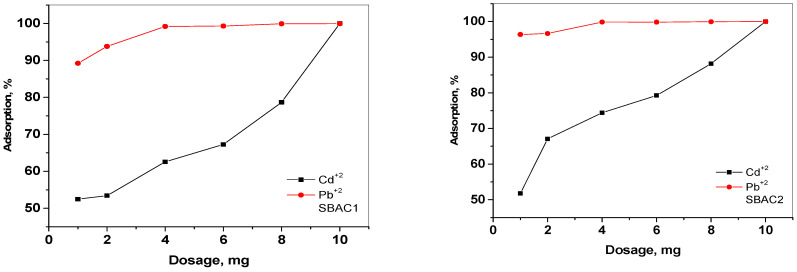
Effect of dosage of SBAC1 and SBAC2 on the adsorption of Cd^+2^ and Pb^+2^; metal ion concentration = 30 mg/L; contact time = 10 min; pH = 7.

The effect of SBAC1 and SBAC2 dosage on the percentage of metal ions adsorbed from the aqueous solution showed that the removal efficiency of metal ions gradually increased with increasing SBAC1 and SBAC2 dosage. As can be seen in Figure 6, increasing the mass of SBAC1 from 1 ppm to 10 ppm increases the percentage of adsorbed metal ions from 52% and 89.233% to 99.997% and 99.977% for Cd^+2^ and Pb^+2^ ions, respectively. An increase in the mass of SBAC2 leads to an increase in the adsorbed percentage of the same ion series from 51.8% and 96.370% to 96.370% and 99.997%. This could be due to the fact that the increase in adsorbent dose provides a larger surface area or more adsorption sites for the metal ions [37].

#### 2.8.2. Influence of the of Metal Ion Concentration

**Figure 7:**
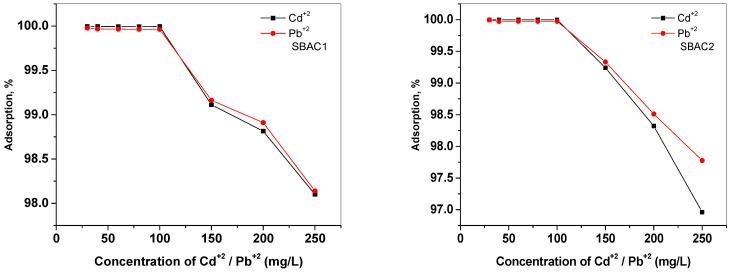
Effect of metal ion concentration on the adsorption by SBAC1 and SBAC2. Adsorbent mass 10 mg; contact time = 10 min; pH = 7 for Cd^+2^ and Pb^+2^.

The effect of a metal ion concentration of 30, 40, 60, 80, 100, 150, 200, and 250 mg/L on the adsorption behavior of SBAC1 and SBAC2 (10 mg/L) was investigated and the results are shown in Figure 7. At 250 ppm, the removal efficiency is 98.1% and 98.14% for Cd^+2^ and Pb^+2^ using SBAC1, but, for SBAC2, the efficiency is 96.96% and 97.77%, respectively. In contrast, at 30 ppm, the efficiency of SBAC1 is 99.99% and 99.97% for Cd^+2^ and Pb^+2^, while SBAC2 has a value of 99.99% for Cd^+2^ and Pb^+2^. This could indicate that the adsorption interaction between the activated carbon and the metal ions is mainly ionic in nature [38]. A high concentration of metal ions limits their transfer to the activated carbon surface and may also be due to the saturation of the active sites of the activated carbon with the metal ions.

#### 2.8.3. Influence of pH

**Figure 8:**
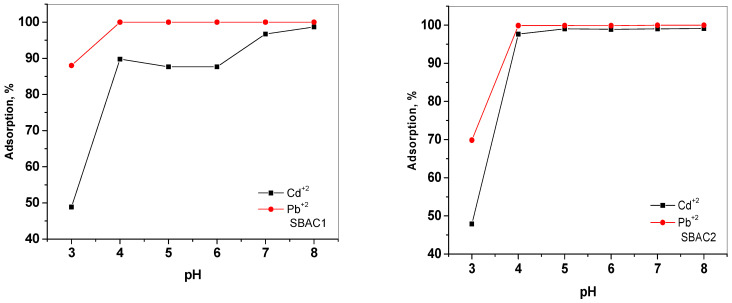
Effect of solution pH on the adsorption of Cd^2+^ and Pb^2+^ by SBAC1 and SBAC2. Adsorbent mass 10 mg, [M]^2+^ = 30 mg/L; contact time = 10 min.

pH is one of the main influences on the adsorption process, especially for heavy metal ions such as Cd^+2^ and Pb^+2^, as they are present in different species depending on the pH [39]. The effect of pH on the adsorption of Cd^+2^ and Pb^+2^ by SBAC1 and SBAC2 was studied in the pH range of 3–8 and the results are shown in Figure 8. It is obvious that the adsorption rate for Cd^+2^ with SBAC1 and SBAC2 gradually increased from 48.87% to 98.69% and 47.86% to 99.14% with increasing the pH from 3 to 8. Moreover, the adsorption percentage for Pb^+2^ with SBAC1 increases abruptly from 88.01 to 99.996 with increasing the pH from 3 to 6, and then remains constant after pH = 6 as the adsorption was already close to 100%, while the adsorption percentage for Pb^+2^ with SBAC2 increases from 69.85 to 99.98 with increasing the pH from 3 to 7, and then remains constant after pH = 7 as the adsorption was already close to 100%. The minimal adsorption observed at low pH values could be due to the fact that the hydrogen ions have a better adsorption than the metal ions due to their higher concentration and mobility [40]. Consequently, the surface of the activated carbon is predominantly covered with H, which prevents metal ions from approaching the binding sites. This is also consistent with the theory of surface complex formation (SCF), as an increase in pH reduces the competition for adsorption sites between protons and metal species, leading to an increase in the adsorption of metal ions [41].

#### 2.8.4. Influence of Time

**Figure 9:**
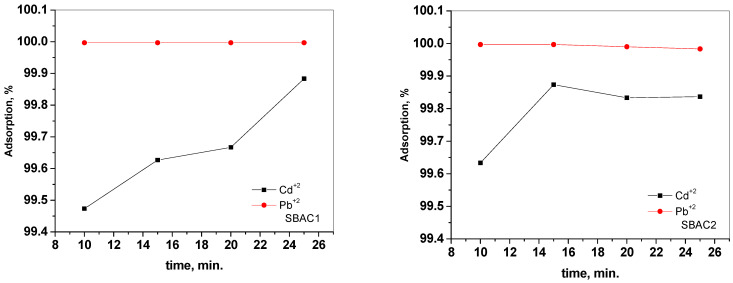
Effect of contact time on the adsorption of Cd^+2^ and Pb^+2^, from aqueous solution by SBAC1 and SBAC2 at pH of 7, 10 ppm activated carbon, and a metal ion concentration of 30 ppm.

The time needed for the interaction between the adsorbate and adsorbent is crucial (i.e., the faster the removal, the better the adsorbent) [40,42]. Hence, it is important to study the effect of contact time on the removal of the target heavy metals with both SBAC1 and the modified SBAC2. Figure 9 shows the effect of contact time on the adsorption of Cd^+2^ and Pb^+2^ onto SBAC1 and SBAC2 from aqueous solutions. In general, the % adsorption of metals ions increased significantly within the first 10 min. Pb^+2^ absorbed completely after 10 min and reached nearly 100% for both SBAC1 and SBAC2. For Cd^+2^, it took approximately 15 min to reach equilibrium with 99.62% adsorption for SBAC1, whereas it reached 99.873% for SBAC2, so SBAC2 is considered a better adsorbent for Cd^+2^.

#### 2.8.5. Influence of Temperature

The influences of temperature on the removal efficiency (%) of Cd^+2^ and Pb^+2^ were tested at different temperatures (40, 50, and 60 °C) at solution pH 7.00. It was noticed that that Cd^+2^ and Pb^+2^ adsorption capacity of SBAC1 and SBAC2 reached its maximum value (99.9%) at 40 °C, while there was a slight drop in the Cd^+2^ and Pb^+2^ adsorption capacity of SBAC1 and SBAC2 at higher temperatures. The adsorption of Cd^2+^ and Pb^2+^ on the SBAC1 and SBAC2 adsorbent at 313, 323, and 333 K was studied to determine how temperature affects adsorption. Equations (1)–(4) were used to calculate thermodynamic parameters such as that change in enthalpy ((∆H^0^), entropy ((∆S^0^), and free energy ((∆G^0^) [43,44,45].

(1) $$\ln K_{c}=\frac{∆S^{0}}{R}-\frac{∆H^{0}}{\mathrm{RT}}$$

(2) $$\ln K_{c}=\frac{q_{e}}{C_{e}}$$

(3) $${∆G}^{0}=-\mathrm{RTln}K_{c}$$

(4) $${∆G}^{0}={∆H}^{0}-T∆S^{0}$$

where K_c_, R, and T (K) are the thermodynamic equilibrium constant (L/g), universal gas constant (8.314 J/mol/K), and absolute solution temperature, respectively.

As seen in Table 5, the ΔH^0^ value of SBAC1 and SBAC2 for absorption Cd^+2^ were −61.2 and −47.64 kJ mol^−1^, indicating an exothermic reaction; thus, the adsorption capacity of SBAC1 and SBAC2 for the adsorption of Cd^+2^ was inhibited under the conditions of a high reaction temperature [43]. Conversely, the ΔH^0^ values of SBAC1 and SBAC2 for the absorption of Pb^+2^ were 217.043 and 141.39 kJ mol^−1^, indicating an endothermic reaction; thus, the Pb^+2^ adsorption capacities of SBAC1 and SBAC2 could be improved under the conditions of a high reaction temperature. Subsequently, during the Cd^+2^ adsorption, a negative ΔS^0^ value for SBAC1 and SBAC2 suggested an improved order of the solid solution interface. On the other hand, during the Pb^+2^ adsorption, all positive ΔS^0^ values of SBAC1 and SBAC2 clearly demonstrated a disordered solid–solution interface [46]. Previous investigations showed that a physical adsorption process was indicated by a Gibbs free energy value (ΔG^0^) between −20 and 0 kJ mol^−1^, while a chemical adsorption process was suggested by ΔG^0^ between −400 and −80 kJ mol^−1^ [47]. At varying temperatures for Cd^+2^ adsorption, the ΔG^0^ values of SBAC1 and SBAC2 ranged from −20 to 0 kJ mol^−1^, suggesting a physical adsorption process caused by either weak electrostatic interaction or Van der Waals force. At all temperatures, the ΔG^0^ values of SBAC1 and SBAC2 ranged from −80 to −20 kJ mol^−1^, suggesting that the physical and chemical composite actions controlled the Pb^+2^ adsorption process of SBAC1 and SBAC2. In addition, as the reaction temperature increased from 313 to 333 K, the ΔG^0^ absolute values of SBAC1 and SBAC2 progressively increased. This outcome demonstrated once more that higher temperatures were helpful in enhancing the ability of SBAC1 and SBAC2 to adsorb lead.

### 2.9. Adsorption Kinetics

**Figure 10:**
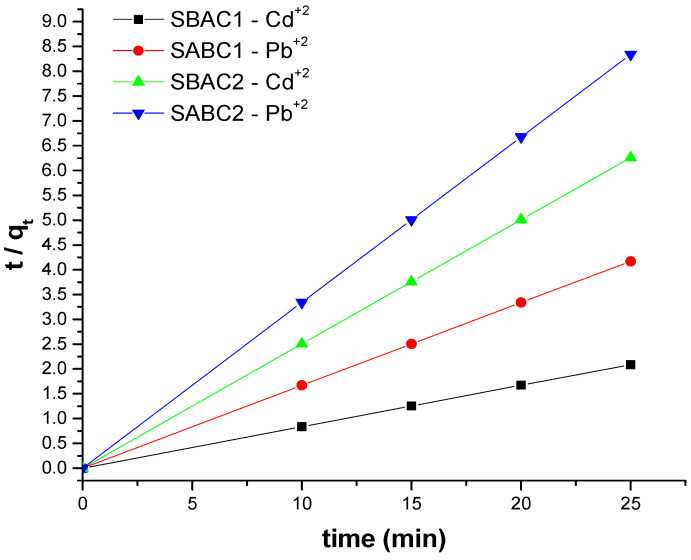
Adsorption kinetics of Cd^+2^ and Pb^+2^ adsorption on SBAC1 and SBAC2, Ci = 30 mg/L, T = 40 °C, and pH =7.00.

The first-order and second-order kinetic models (Equations (6) and (7)) were used to calculate the kinetic parameters of Cd^+2^ and Pb^+2^ adsorption on the SBAC1 and SBAC2 adsorbent. The adsorption kinetics of the first-order and second-order kinetic models are depicted in Figure 10. As shown in Table 4, the second-order kinetic model appropriates the experimental results more effectively than the first-order kinetic model. Furthermore, the theoretical maximum adsorption at equilibrium predicted by the second-order model agreed well with the experimental result values observed. As a result, the second-order kinetic model proved appropriate for describing the kinetics of Cd^+2^ and Pb^+2^ adsorption on the SBAC1 and SBAC2 absorbent [48]. The coefficients of the adsorption kinetic model variables derived using the statistical program (version 7) were shown in Table 6.

### 2.10. Adsorption Isotherm

**Figure 11:**
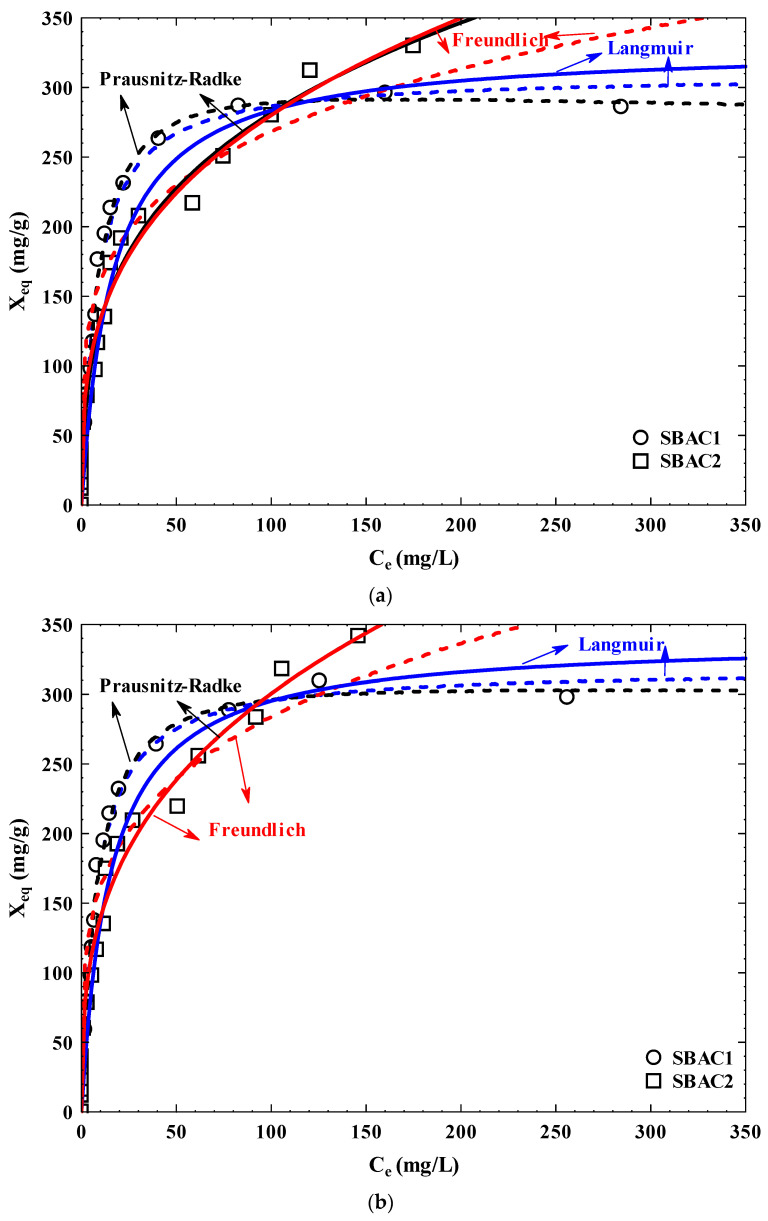
Adsorption isotherms of (**a**) Cd^+2^ and (**b**) Pb^+2^ on both activated carbons SBAC1 and SBAC2 at carbon mass 100 mg, solution pH = 7, and T = 25 °C.

Figure 11 illustrates the adsorption isotherms of Cd^+2^ and Pb^+2^ on SBAC1 and SBAC2 at T = 25 °C and solution pH = 7. Giles classified both of the adsorption isotherms as having L-behavior [49]. When the ratio of the pollutants’ adsorbed fraction on carbon material to their residual concentration in the aqueous solution decreases, a concave curve appears. Furthermore, as suggested, this behavior showed a significant attraction between the adsorbate molecules and the adsorbent surface as recommended by similar studies related to the adsorption of organic and inorganic pollutants from aqueous solution [50,51,52].

To the experimental results, the Langmuir, Freundlich, and Prausnitz–Radke isotherm models have been applied. As shown in Table 7, the adsorption capacity of SBAC2 for both pollutants is higher than SBAC1, which may be attributed to the high surface area for SBAC2 (498.39 m^2^/g) than that for SBAC1 (336.34 m^2^/g) and high total pore volume for SBAC2 (0.37 cm^3^/g) than that of SBAC1 (0.27 cm^3^/g), and a high oxygen content in SBAC2 (21.93%) than that for SBAC1 (19.80%). Moreover, the adsorption capacity of Pb^+2^ and the Cd^+2^ on both activated carbons was similar due to a similar atomic radius of Pb^+2^ (1.75 Å) and Cd^+2^ (1.54 Å) [53]. The Prausnitz–Radke model was the best model to describe the adsoption isotherm for all cases; moreover, it had a slightly better fit than the Langmuir model, followed by the Freundlich model, to the adsorption data of Cd^+2^ and Pb^+2^ on SBAC1, with %D for the Prausnitz–Radke model being 5.80 and 6.66, and that for the Langmuir model being 5.49 and 6.67, respectively. That means the contribution of monolayer adsorption on SBAC1 for both pollutants was higher than the multilayer adsorption process, whereas, in the case of SBAC2, it had a slightly better fit than the Freundlich model, followed by the Langmuir model, to the adsorption data of Cd^+2^ and Pb^+2^ on SBAC1, with %D for the Prausnitz–Radke model being 8.41 and 8.50 and that for the Freundlich model being 9.41 and 8.49, respectively. That means the contribution of multilayer adsorption on SBAC2 for both pollutants was higher than the monolayer adsorption process. This is confirmed by the fact that SBAC2 had more adsorption capacity than that for SBAC1 for both heavy metals.

### 2.11. Analysis of Real Wastewater

The real wastewater samples were collected from six different locations in Bisha, Saudi Arabia to verify the applicability of SBAC1 and SBAC2 for the removal of heavy metals (Cd^+2^ and Pb^+2^) from real water samples. The mean value of percentage removal of Cd^+2^ and Pb^+2^ from SBAC1 and SBAC2 is shown in Table 8. The proposed methods were successfully used to remove Cd^+2^ and Pb^+2^ from some environmental and industrial wastewater samples. The results in this table show that the proposed methods were effective and acceptable for the removal of Cd^+2^ and Pb^+2^ from water samples, with a high removal efficiency (%) ranging from 97.00% to 99.80%.

### 2.12. Recovery and Recyclability SBAC1 and SBAC2

**Figure 12:**
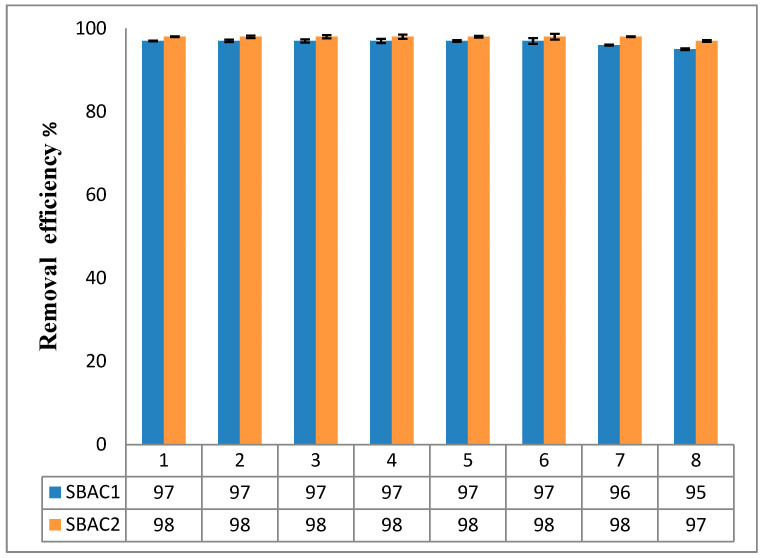
The removal efficiency of Cd^+2^ and Pb^+2^ onto intact and regenerated SBAC1 and SBAC2 adsorbent during eight adsorption/desorption cycles.

The prospect of reusing the recovered SBAC1 and SBAC2 in successive cycles was investigated with the aim of learning more about the environmentally and economically beneficial properties. By reusing the adsorbent loaded with Cd^+2^ and Pb^+2^ after SBAC1 and SBAC2 were swirled in ethanol for 5 h, washed with ethanol and dried at 50 °C for 5 h (thermal desorption in a muffle furnace), the regeneration of SBAC1 and SBAC2 adsorbent was investigated. Eight times the loaded adsorbent was subjected to Cd^+2^ and Pb^+2^ adsorption under optimal renewal conditions. Figure 12 shows the percentage Cd^+2^ and Pb^+2^ removal rate for the eight reuses, indicating that SBAC1 and SBAC2 could be recycled for six consecutive cycles without losing their adsorption capacity. However, in the two subsequent runs (7 and 8) under the same conditions, a reduced adsorption capacity was observed. This indicates that SBAC1 and SBAC2 are trustworthy, and the excellent recycling results support the sparing use of the adsorbent in the purification of water from Cd^+2^ and Pb^+2^.

### 2.13. Comparison with Another Method

From the data listed in Table 9, the high efficiency of SBAC1 and SBAC2 and its modification for removal of the Cd^+2^ and Pb^+2^ ions from the working samples are generally revealed.

## 3. Materials and Methods

### 3.1. Chemicals

Analytical chemical reagents (ARs), which were already completely exhausted, were used without further purification. Sodium hydroxide (99%), cadmium chloride ≥ 99.9%, hydrochloric acid (99%), hydrogen peroxide ≥ 99.8, and lead acetate (≥99.9%) were purchased from Aldrich Chemical Co. (Taufkirchen, Germany). Milli-Q^®^ devices (Millipore, Sofia, Bulgaria) provided ultrapure water for all of the solutions.

### 3.2. Instruments

The concentration of Cd^+2^ and Pb^+2^ was estimated using ICP-Optical Emission Spectroscopy (PerkinElmer Avio^®^ 220 Max ICP-OES, Shelton, CT, USA). Hanna pH-meter (Benchtop pH/ORP Meter—HI2211, Woonsocket, RI, USA) was used for pH readings. Digital hotplate stirrer (Model MSH-20D) was manufactured by DAIHAN Scientific Co., Ltd. Wonju-si, Republic of Korea. An elemental analyzer (Elementar, Langenselbold, Germany) was used for the elemental analysis (CHNS) of the prepared activated carbon. The specific surface area and pore structure of the prepared activated carbons were measured using a surface area analyzer (Quantachrome, Boynton Beach, FL, USA) and nitrogen adsorption at 196 °C. The Brunauer–Emmett–Teller (BET) method was used to calculate the surface area (SBET) of the activated carbons produced. Powder X-ray diffraction (XRD) was performed on the manufactured products using an 18 kW diffractometer (Bruker, Billerica, MA, USA; model D8 Advance) and monochromatic Cu-Ka radiation. Images were acquired using a field emission scanning electron microscope (FE-SEM) and a JEOL JSM-6500F microscope (Peabody, MA, USA). HR-TEM images were acquired using a JEM-2100 microscope with an accelerating voltage of 200 kV. The samples were ultrasonically dispersed in ethanol on a copper grid. The zeta potential was determined using a Zetasizer Nano series instrument (Nano ZS, Malvern, UK). Fourier-transform infrared spectroscopy (Thermo Scientific NicoletTM iS10, Waltham, MA, USA) recorded at a wavenumber of 400–4000 cm^−1^ was used to investigate the surface functional groups of the prepared activated carbon.

### 3.3. Fabrication of Activated Carbon

Activated carbon is produced from two types of raw sewage: thickened samples SBAC1 and unthickened samples SBAC2. In this process, the raw sewage sludge is taken from a drying bed of a wastewater treatment plant in Bisha, in the southwestern Saudi Arabian province of Asir. The sewage sludge was dried in an oven at 105 °C and ground into smaller particles with a diameter of 0.75 mm. A chemical activating agent, KOH, is used in the activation process. During production, the sewage sludge samples are first carbonized for 2 h at 623 K (350 °C) at a heating rate of 10 °C/min. The samples are then impregnated with the activating agent (KOH) for two days at 333 K (60 °C), and then pyrolyzed for two hours at 573 K (300 °C), and then for one hour at 1173 K (900 °C). To get rid of the potassium hydroxide from the generated ACs, the samples were rinsed with 0.10 M HCl, and then with distilled water until no chloride ions could be detected in the water used for washing. In addition, the carbon particles were soaked in H_2_O_2_ for three days at room temperature with constant stirring. They were then filtered and rinsed with distilled water until the pH of the solution remained constant. After washing, the samples were air-dried at 383 K (110 °C) for 24 h [57].

### 3.4. Adsorption Studies

#### 3.4.1. Adsorption Procedures of Cd^+2^ and Pb^+2^

First, 25 mL aqueous solutions of Cd^+2^ and Pb^+2^ ions (5, 10, 15, 20, 25, and 30 mg/L) were made and their values of pH were detected using 0.1 N HCl/NaOH solution. The adsorption studies were conducted in Erlenmeyer flasks holding the solution of metal at the indicated starting concentration (30 mg/L). The mixture of the metal solution and the activated carbon adsorbent was kept at 30 °C for 30 min with constant stirring. The adsorbent was separated using a centrifuge (5000 rpm) and the filtrate was analyzed by inductively coupled plasma (ICP) optical emission spectroscopy, it is about the determination of the Cd^+2^ and Pb^+2^ ion concentration. A series of experimental parameters such as metal ion concentration (5–30 mg/L), adsorbent dose (10–50 mg), pH (3–8), temperature (40–80 °C), and contact time (0–30 min) were checked to evaluate the optimal conditions for the maximum removal efficiency (%) of Cd^+2^ and Pb^+2^ from aqueous solution via the adsorbents used [56,58,59,60]. All experiments were carried out in triplicate and the mean values were given. The elimination efficiency (%) of the studied metal ion was estimated using the following equation:

Elimination efficiency (%) = (C_o_ − C_e_)/C_o_ × 100 (5)

where C_o_: the starting concentration of the metal ion and C_e_: the residual concentration of the metal ion after 30 min.

#### 3.4.2. Adsorption Isotherms

The primary concentrations of Cd^+2^ and Pb^+2^ used to estimate the adsorption isotherms of both SBAC1 and SBAC2 were between 30 and 1000 mg/L. Equations (6)–(8) reproduce the adsorption isotherm models of Langmuir, Freundlich, and Prausnitz–Radke. The previous models represent the most common models of the adsorption isotherm applied to define the adsorption mechanism and type:

(6) $$X_{\mathrm{eq}}=\frac{BX_{m}C_{e}}{1+BC_{e}}$$

(7) Xeq=KFCe1nF

(8) $$q=\frac{aC_{A}}{1+b{C_{A}}^{\beta }}$$

where X_eq_ is the adsorption yield (mg/g), X_m_ is the adsorption capacity (mg/g), B is the constant of Langmuir yield (L/mg), C_e_ is the contaminant equilibrium concentration (mg/L), 1/n_F_ is the heterogeneity of the SBAC1 and SBAC2 surface, K_F_ is the relative adsorption capacity, and a (L/g), b (L^β^/mg^β^), and β are the constants.

Equation (9) is used to compute the average absolute percentage deviations for all models of adsorption isotherms.

(9) $$\%D=\frac{1}{N}\sum_{i=1}^{N}\left|\frac{X_{\exp}-X_{\mathrm{pred}}}{X_{\exp}}\right|\times \;100\%$$

where %D: percentage deviation, N: experiments number, X_exp_: experimental adsorption yield, and X_Pred_: predicted adsorption yield.

#### 3.4.3. Adsorption Kinetics

According to the previous adsorption procedures, Equations (10) and (11) showed the 1st- and 2nd-order kinetic equations that reflect the most commonly used adsorption kinetic [57].

(10) $$q=q_{\mathrm{pred},1}\left(1-e^{-k_{1}t}\right)$$

(11) $$q=\frac{{q_{\mathrm{pred},2}^{2}k}_{2}t}{1+{q_{\mathrm{pred},2}k}_{2}t}$$

where: q: weight of adsorbed adsorbate, mg/g, q_pred,1_: weight of adsorbed adsorbate expected from the 1st-order kinetic model, mg/g, q_pred,2_: weight of adsorbed adsorbate expected from the 2nd-order kinetic model, mg/g, k_1_: the 1st-order kinetic model rate constant, 1/min, k_2_: the 2nd-order kinetic model rate constant, g/mg/min, and t: time, min.

### 3.5. Environmental and Industrial Wastewater Samples

A variety of environmental water samples, including tap water (TW), groundwater (GW), Red Sea water (RSW), wastewater from the Bisha environmental treatment plant before the biological treatment stage (BT), and other wastewater samples (WW1 and WW2) (from different regions in Bisha, Saudi Arabia), were collected and filtered through Whitman No. 40 filter paper to remove all undissolved particles. A 1000 mL sample of each water sample was placed in a 1000 mL glass beaker and boiled until the volume was reduced to 50 mL. The remaining residue was then treated with 2 mL of 70% HClO_4_ and 5 mL of concentrated nitric acid, and the sample was carefully heated for 30 min until dense white vapors appeared. The sample was cooled and 10 mL of deionized water was added. It was then boiled again for 30 min until the vapors were completely released [61]. The samples were then cooled, filtered, and taken to ICP to determine the starting concentration of Cd^+2^ and Pb^+2^ in these samples. The previous processes to remove Cd^+2^ and Pb^+2^ from the water samples were repeated and the remaining Cd^+2^ and Pb^+2^ concentrations were measured by ICP.

## 4. Conclusions

Activated carbon (SBAC1/SBAC2) derived from sewage sludge waste was used as an adsorbent for the effective removal of Cd^+2^ and Pb^+2^ from an aqueous solution. The structural properties, functional groups, and morphological structures of the prepared activated carbon adsorbent were determined by CHNS, BET, FT-IR, XRD, XRF, SEM, TEM, and zeta potential. The adsorption isotherm model was best described by Freundlich (SBAC1) and Langmuir (SBAC2) isotherms, with maximum adsorption capacities of 309.24, 329.62, 318.46, and 339.61 mg/g at pH 7 for Cd^+2^ and Pb^+2^, respectively. The kinetic model was applied to the adsorption equilibrium data to predict the adsorption mechanism of the adsorbent. The result showed that the adsorption followed the pseudo-second-order kinetic model. The thermodynamic parameters of adsorption showed that the adsorption of Cd^+2^ and Pb^+2^ proceeded spontaneously and were endothermic and that the randomness during the adsorption process increased with increasing temperature. In addition, the development of the prepared activated carbon as an adsorbent could be successfully used for the treatment of water containing Cd^+2^ and Pb^+2^ ions due to its cost-efficiency, reusability, large surface area, and high adsorption capacity.

## Figures and Tables

**Table 1:** Ultimate analysis of raw sewage sludge (RSS), SBAC1, and SBAC2.

Samples	Yield %	C (wt. %) ^a^	H (wt. %) ^a^	O (wt. %) ^b^	N (wt. %)	H/C	O/C	N/C	Ash %
RSS		67.7	9.2	14.3	9.3	0.14	0.21	0.14	13.9
SBAC1	83	90.8	4.2	5.1	1.6	0.05	0.06	0.02	7.4
SBAC1	85	93.1	5.5	4.9	2.1	0.06	0.05	0.02	2.7

^a^ = obtained by elemental analysis. ^b^ = computed using the mass conservation law difference.

**Table 2:** The results of XRF analysis of SBAC1 and SBAC2.

Component	Elements (wt %)		
	RSS	SBAC1	SBAC2
Al	3.00	3.65	5.08
Ca	11.21	19.01	20.67
Cu	0.01	0.04	0.03
Fe	8.02	9.44	9.52
K	1.03	3.69	1.56
Mn	0.14	0.24	0.22
Nb	0.01	0.04	0.02
Ni	0.01	0.03	0.02
P	4.08	4.93	5.56
Si	13.20	15.20	15.05
Ti	1.34	1.76	1.68
Sr	0.12	0.15	0.22
Zn	0.35	0.59	0.45
Zr	0.10	0.03	0.02
Co	0.04	0.06	—
Cl	1.01	1.73	—
Mo	0.02	0.04	—

**Table 3:** The porous structure properties of SBAC1 and SBAC2.

Samples	S_BET_ (m^2^ g^−1^)	V_T_ (cm^3^ g^−1^)	V_BJH_ (cm^3^ g^−1^)	ζ (mV)
SBAC1	336.339	0.268858	1.59873	−11.50
SBAC2	498.386	0.374884	1.5026	−8.85

**Table 4:** Microcrystalline structure of SBAC1 and SBAC2.

Samples	2θd_100_ (°)	FWHM (β°)	hkl	d_100_ (nm)	$L_{c}^{100}(nm)$
SBACI	26.45	0.4942	100	14.7195	16.2498
	31.80	0.4544	002	17.7801	
SBAC2	28.31	0.5428	100	15.0883	16.5584
	31.80	0.4580	002	18.0286	

**Table 5:** Thermodynamic parameters for the adsorption of Pb^+2^ and Cd^+2^ on SBAC1 and SBAC2.

Sample	T/K	ΔG^0^ (kJ mol^−1^)		ΔH^0^ (kJ mol^−1^)		ΔS^0^ (J K^−1^·mol^−1^)	
		Cd^+2^	Pb^+2^	Cd^+2^	Pb^+2^	Cd^+2^	Pb^+2^
	313	−15.06	−24.55				
SBAC1	323	−12.92	−25.82	−61.24	217.04	−0.1475	0. 7718
	333	−11.58	−26.00				
	313	−15.50	−25.44				
SBAC2	323	−14.61	−26.00	−47.64	141.39	−0.1027	0.5330
	333	−13.45	−27.04				

**Table 6:** Results obtained from applying first-order and second-order kinetic models to the Cd^+2^ and Pb^+2^ adsorption experimental data.

Kinetics Models	Variables	SBAC1		SBAC2	
		Pb^+2^	Cd^+2^	Pb^+2^	Cd^+2^
Pseudo-first-order	k_1_ (min^−1^)	0.00032	0.00085	0.0009	0.0009
	q_e(cal)_ (mg g^−1^)	12.00	11.937	12.00	11.9848
	R_1_^2^	0.500	0.1529	0.6621	0.6619
	q_e(exp)_ (mg g^−1^)	1.7716	1.249	1.471	1.4720
Pseudo-second-order	k_2_ [g mg^−1^ min^−1^]	6.94	4.3556	−110.1	13.527
	q_e(cal)_ (mg g^−1^)	11.999	11.936	11.998	11.9848
	R_2_^2^	1.00	1.00	1.00	1.00
	q_e(exp)_ (mg g^−1^)	11.999	11.978	11.998	11.9817

**Table 7:** Adsorption parameters determined using the three adsorption isotherm models of Cd^+2^ and Pb^+2^ on both activated carbons SBAC1 and SBAC2.

AC	Pollutant	Langmuir					Freundlich			Prausnitz–Radke			
		X_m_ ^(a)^ (mg/g)	B ^(b)^ (L/mg)	BX_m_ ^(c)^ (L/g)	X′_m_ × 10^−4^ (mg/m^2^/g)	%D	K_F_ ^(d)^ (L/g)	1/n_F_ ^(e)^	%D	a ^(f)^ (L/g)	b ^(g)^ (L^β^/mg^β^)	β ^(h)^	%D
SBAC 1	Cd^+2^	309.24	0.13	40.20	0.92	5.94	96.07	0.22	23.51	33.37	0.08	1.06	5.80
SBAC 2		329.62	0.06	19.78	0.66	12.84	63.79	0.32	9.41	109.99	1.42	0.71	8.41
SBAC 1	Pb^+2^	318.46	0.13	41.40	0.95	6.67	91.66	0.25	21.70	37.06	0.10	1.03	6.66
SBAC 2		339.61	0.07	23.77	0.68	13.19	64.79	0.33	8.49	1.52 × 10^7^	2.34 × 10^5^	0.67	8.50

Note: ^(a)^ Xm: capacity of adsorption (mg/g); ^(b)^ B: constant (L/mg); ^(c)^ BXm: adsorbent–adsorbate relative affinity (L/g); ^(d)^ KF: relative capacity for adsorption (L/g); ^(e)^ 1/nF: sorption intensity or surface heterogeneity; ^(f)^ a: constant (L/g); ^(g)^ b: constant (Lβ/mgβ); and ^(h)^ β: constant.

**Table 8:** Removal of the target heavy metal ions from spiked real sample by SBAC1 and SBAC2.

Sample	pH	Metal Ions	% Adsorption	
			SBAC1	SBAC2
TW	7.50	Cd^+2^	99.00 ± 0.01	99.44 ± 0.02
		Pb^+2^	99.03 ± 0.01	99.80 ± 0.003
GW	8.00	Cd^+2^	98.02 ± 0.04	99.60 ± 0.005
		Pb^+2^	98.53 ± 0.05	98.73 ± 0.006
RSW	7.95	Cd^+2^	98.33 ± 0.03	98.58 ± 0.004
		Pb^+2^	98.58 ± 0.01	98.88 ± 0.02
BTW	7.27	Cd^+2^	97.00 ± 0.02	98.00 ± 0.001
		Pb^+2^	97.19 ± 0.03	98.50 ± 0.001
WW1	6.91	Cd^+2^	97.33 ± 0.01	98.44 ± 0.03
		Pb^+2^	97.96 ± 0.06	98.77 ± 0.01
WW2	7.45	Cd^+2^	97.60 ± 0.02	97.74 ± 0.001
		Pb^+2^	97.54 ± 0.01	97.88 ± 0.003

**Table 9:** Comparison between the maximum adsorption capacity of the target divalent metal ions by SBAC1 and SBAC2 and other adsorbents.

Adsorbent	Adsorbent Dosage (g)	Metal Ions	Lower/Upper Concentration (mg/L)	pH	Time	q_m_ (mg/g)	Reference
Zeolite	0.3 0.005	Pb^+2^ Cd^+2^	5–20	6 4	24 h 20 min	56.82 50.2	[39] [54]
MWCNTs	0.03 0.05	Pb^+2^ Cd^+2^	3–250 50–150	4 8	1.5 h 1 h	200 200	[39] [55]
Activated carbon	0.02 0.02	Pb^+2^ Cd^+2^	30–200 30–200	2 2	24 h 24 h	294.11 178.5	[56]
SBAC1	0.02	Pb^+2^ Cd^+2^	30–250 30–250	7 7	30 min 30 min	318.46 309.24	This work
SBAC2	0.02	Pb^+2^ Cd^+2^	30–250 30–250	7 7	30 min 30 min	339.61 329.62	This work

## Data Availability

The datasets used and/or analyzed during the current study are available from the corresponding author upon reasonable request.

## References

[1] Kacprzak M., Neczaj E., Fijałkowski K., Grobelak A., Grosser A., Worwag M., Rorat A., Brattebo H., Almås Å., Singh B.R. (2017). Sewage sludge disposal strategies for sustainable development. Environ. Res..

[2] Almahbashi N.M.Y., Kutty S.R.M., Ayoub M., Noor A., Salihi I.U., Al-Nini A., Jagaba A.H., Aldhawi B.N.S., Ghaleb A.A.S. (2021). Optimization of Preparation Conditions of Sewage sludge based Activated Carbon. Ain Shams Eng. J..

[3] Spinosa L., Ayol A., Baudez J.-C., Canziani R., Jenicek P., Leonard A., Rulkens W., Xu G., Van Dijk L. (2011). Sustainable and Innovative Solutions for Sewage Sludge Management. Water.

[4] Bibby K., Peccia J. (2013). Identification of Viral Pathogen Diversity in Sewage Sludge by Metagenome Analysis. Environ. Sci. Technol..

[5] Nnorom M.-A., Saroj D., Avery L., Hough R., Guo B. (2023). A review of the impact of conductive materials on antibiotic resistance genes during the anaerobic digestion of sewage sludge and animal manure. J. Hazard. Mater..

[6] Samolada M.C., Zabaniotou A.A. (2014). Comparative assessment of municipal sewage sludge incineration, gasification and pyrolysis for a sustainable sludge-to-energy management in Greece. Waste Manag..

[7] del Rosario Moreno V.M., Omar Francisco González V., Virginia Hernández M., Rigoberto Tovar G., Hosam El-Din M.S., Refaat F.A. (2018). Removal of Heavy Metals Using Adsorption Processes Subject to an External Magnetic Field. Heavy Metals.

[8] Senthilkumar T.S., Chattopadhyay S.K., Miranda L.R. (2017). Optimization of Activated Carbon Preparation from Pomegranate Peel (Punica granatum Peel) Using RSM. Chem. Eng. Commun..

[9] Wen Q., Li C., Cai Z., Zhang W., Gao H., Chen L., Zeng G., Shu X., Zhao Y. (2011). Study on activated carbon derived from sewage sludge for adsorption of gaseous formaldehyde. Bioresour. Technol..

[10] Im U.-S., Kim J., Lee S.H., Lee S.m., Lee B.-R., Peck D.-H., Jung D.-H. (2019). Preparation of activated carbon from needle coke via two-stage steam activation process. Mater. Lett..

[11] Sulaiman N.S., Hashim R., Mohamad Amini M.H., Danish M., Sulaiman O. (2018). Optimization of activated carbon preparation from cassava stem using response surface methodology on surface area and yield. J. Clean. Prod..

[12] Chen X., Jeyaseelan S., Graham N. (2002). Physical and chemical properties study of the activated carbon made from sewage sludge. Waste Manag..

[13] Devi P., Saroha A.K. (2017). Utilization of sludge based adsorbents for the removal of various pollutants: A review. Sci. Total Environ..

[14] Björklund K., Li L.Y. (2017). Adsorption of organic stormwater pollutants onto activated carbon from sewage sludge. J. Environ. Manag..

[15] N’goran K.P.D.A., Diabaté D., Yao K.M., Kouassi N.G.L.B., Gnonsoro U.P., Kinimo K.C., Trokourey A. (2018). Lead and cadmium removal from natural freshwater using mixed activated carbons from cashew and shea nut shells. Arab. J. Geosci..

[16] Kavand M., Eslami P., Razeh L. (2020). The adsorption of cadmium and lead ions from the synthesis wastewater with the activated carbon: Optimization of the single and binary systems. J. Water Process Eng..

[17] Gerçel Ö., Gerçel H.F. (2007). Adsorption of lead(II) ions from aqueous solutions by activated carbon prepared from biomass plant material of Euphorbia rigida. Chem. Eng. J..

[18] Thornton I., Butler D., Docx P., Hession M., Makropoulos C., McMullen M., Nieuwenhuijsen M., Pitman A., Rautiu R., Sawyer R. (2001). Pollutants in urban waste water and sewage sludge. Final Report Prepared for European Commission Directorate-General Environment.

[19] Asuquo E., Martin A., Nzerem P., Siperstein F., Fan X. (2017). Adsorption of Cd(II) and Pb(II) ions from aqueous solutions using mesoporous activated carbon adsorbent: Equilibrium, kinetics and characterisation studies. J. Environ. Chem. Eng..

[20] Dzul Erosa M.S., Saucedo Medina T.I., Navarro Mendoza R., Avila Rodriguez M., Guibal E. (2001). Cadmium sorption on chitosan sorbents: Kinetic and equilibrium studies. Hydrometallurgy.

[21] Gascó G., Lobo M.C. (2007). Composition of a Spanish sewage sludge and effects on treated soil and olive trees. Waste Manag..

[22] Gascó G., Blanco C.G., Guerrero F., Méndez Lázaro A.M. (2005). The influence of organic matter on sewage sludge pyrolysis. J. Anal. Appl. Pyrolysis.

[23] Cao J.P., Xiao X.B., Zhang S.Y., Zhao X.Y., Sato K., Ogawa Y., Wei X.Y., Takarada T. (2011). Preparation and characterization of bio-oils from internally circulating fluidized-bed pyrolyses of municipal, livestock, and wood waste. Bioresour. Technol..

[24] Chen Z., Hu M., Cui B., Liu S., Guo D., Xiao B. (2016). The effect of bioleaching on sewage sludge pyrolysis. Waste Manag..

[25] Kim S.-H., Kim S., Yoom H., Son H. (2022). Evaluation of organics reduction performance of the GAC filtration with regenerated carbons using the long-term operational data of drinking water treatment facilities. AQUA—Water Infrastruct. Ecosyst. Soc..

[26] Park J.E., Lee G.B., Hong B.U., Hwang S.Y. (2019). Regeneration of Activated Carbons Spent by Waste Water Treatment Using KOH Chemical Activation. Appl. Sci..

[27] Reig F.B., Adelantado J.G., Moreno M.M. (2002). FTIR quantitative analysis of calcium carbonate (calcite) and silica (quartz) mixtures using the constant ratio method. Application to geological samples. Talanta.

[28] Kenne Diffo B., Elimbi A., Cyr M., Dika Manga J., Tchakoute Kouamo H. (2015). Effect of the rate of calcination of kaolin on the properties of metakaolin-based geopolymers. J. Asian Ceram. Soc..

[29] Yang B., Liu Y., Liang Q., Chen M., Ma L., Li L., Liu Q., Tu W., Lan D., Chen Y. (2019). Evaluation of activated carbon synthesized by one-stage and two-stage co-pyrolysis from sludge and coconut shell. Ecotoxicol. Environ. Saf..

[30] Herselman J., Snyman H. (2009). Guidelines for the utilisation and disposal of wastewater sludge. Water Research Commission.

[31] Al-Malack M.H., Dauda M. (2017). Competitive adsorption of cadmium and phenol on activated carbon produced from municipal sludge. J. Environ. Chem. Eng..

[32] Ahmad A., Idris A. (2014). Preparation and characterization of activated carbons derived from bio-solid: A review. Desalination Water Treat..

[33] Jindarom C., Meeyoo V., Kitiyanan B., Rirksomboon T., Rangsunvigit P. (2007). Surface characterization and dye adsorptive capacities of char obtained from pyrolysis/gasification of sewage sludge. Chem. Eng. J..

[34] Boehm H.P. (2002). Surface oxides on carbon and their analysis: A critical assessment. Carbon.

[35] Keppetipola N.M., Dissanayake M., Dissanayake P., Karunarathne B., Dourges M.A., Talaga D., Servant L., Olivier C., Toupance T., Uchida S. (2021). Graphite-type activated carbon from coconut shell: A natural source for eco-friendly non-volatile storage devices. RSC Adv..

[36] Everett D.H., Stone F.S. (1958). The Structure and Properties of Porous Materials.

[37] Rengaraj S., Moon S.-H. (2002). Kinetics of adsorption of Co (II) removal from water and wastewater by ion exchange resins. Water Res..

[38] Elsehly E., Chechenin N., Makunin A., Vorobyeva E., Motaweh H. (2015). Oxidized carbon nanotubes filters for iron removal from aqueous solutions. Int. J. New Technol. Sci. Eng..

[39] Salam E.A., Abou El-Nour K.M., Awad A.A., Orabi A.S. (2020). Carbon nanotubes modified with 5,7-dinitro-8-quinolinol as potentially applicable tool for efficient removal of industrial wastewater pollutants. Arab. J. Chem..

[40] Kosa S.A., Al-Zhrani G., Salam M.A. (2012). Removal of heavy metals from aqueous solutions by multi-walled carbon nanotubes modified with 8-hydroxyquinoline. Chem. Eng. J..

[41] Dzombak D.A., Morel F.M. (1991). Surface Complexation Modeling: Hydrous Ferric Oxide.

[42] Mushtaq S., Jamil F., Hussain M., Inayat A., Majeed K., Akhter P., Khurram M.S., Shanableh A., Kim Y.M., Park Y.-K. (2024). Utilizing sludge-based activated carbon for targeted leachate mitigation in wastewater treatment. Environ. Res..

[43] Zhou J., Zhang Z., Cheng B., Yu J. (2012). Glycine-assisted hydrothermal synthesis and adsorption properties of crosslinked porous α-Fe_2_O_3_ nanomaterials for p-nitrophenol. Chem. Eng. J..

[44] Luo C., Tian Z., Yang B., Zhang L., Yan S. (2013). Manganese dioxide/iron oxide/acid oxidized multi-walled carbon nanotube magnetic nanocomposite for enhanced hexavalent chromium removal. Chem. Eng. J..

[45] Nassar M.Y., Ahmed I.S., Mohamed T.Y., Khatab M. (2016). A controlled, template-free, and hydrothermal synthesis route to sphere-like α-Fe2O3 nanostructures for textile dye removal. RSC Adv..

[46] Anastopoulos I., Massas I., Ehaliotis C. (2013). Composting improves biosorption of Pb^2+^ and Ni^2+^ by renewable lignocellulosic materials. Characteristics and mechanisms involved. Chem. Eng. J..

[47] Liu Q.-S., Zheng T., Wang P., Jiang J.-P., Li N. (2010). Adsorption isotherm, kinetic and mechanism studies of some substituted phenols on activated carbon fibers. Chem. Eng. J..

[48] Li Q., Yang F., Zhang J., Zhou C. (2020). Magnetic Fe_3_O_4_/MnO_2_ core–shell nano-composite for removal of heavy metals from wastewater. SN Appl. Sci..

[49] Giles C.H., Smith D., Huitson A. (1974). A general treatment and classification of the solute adsorption isotherm. I. Theoretical. J. Colloid Interface Sci..

[50] Alrowais R., Said N., Bashir M.T., Ghazy A., Alwushayh B., Daiem M.M.A. (2023). Adsorption of Diphenolic Acid from Contaminated Water onto Commercial and Prepared Activated Carbons from Wheat Straw. Water.

[51] Alrowais R., Bashir M.T., Khan A.A., Bashir M., Abbas I., Abdel Daiem M.M. (2024). Adsorption and Kinetics Modelling for Chromium (Cr^6+^) Uptake from Contaminated Water by Quaternized Date Palm Waste. Water.

[52] Alrowais R., Abdel Daiem M.M., Nasef B.M., Said N. (2023). Activated Carbon Fabricated from Biomass for Adsorption/Bio-Adsorption of 2,4-D and MCPA: Kinetics, Isotherms, and Artificial Neural Network Modeling. Sustainability.

[53] Sulaymon A.H., Mohammed A.A., Al-Musawi T.J. (2013). Competitive biosorption of lead, cadmium, copper, and arsenic ions using algae. Environ. Sci. Pollut. Res..

[54] Samadi S., Shalmani M.M., Zakaria S.A. (2019). Removal of heavy metals from Tehran south agricultural water by Zeolite N.P./PEG/GO nano-composite. J. Water Environ. Nanotechnol..

[55] Obayomi K., Bello J., Yahya M.D., Chukwunedum E., Adeoye J. (2020). Statistical analyses on effective removal of cadmium and hexavalent chromium ions by multiwall carbon nanotubes (MWCNTs). Heliyon.

[56] Karnib M., Kabbani A., Holail H., Olama Z. (2014). Heavy metals removal using activated carbon, silica and silica activated carbon composite. Energy Procedia.

[57] Méndez-Díaz J.D., Rivera-Utrilla J., Sánchez-Polo M., Bautista-Toledo I. (2012). Adsorption/bioadsorption of phthalic acid, an organic micropollutant present in landfill leachates, on activated carbons. J. Colloid Interface Sci..

[58] Ullah M., Nazir R., Khan M., Khan W., Shah M., Afridi S.G., Zada A. (2019). The effective removal of heavy metals from water by activated carbon adsorbents of Albizia lebbeck and Melia azedarach seed shells. Soil Water Res..

[59] Wilson N. (2020). Soil water monitoring devices. Soil Water and Ground Water Sampling.

[60] Adam A.M., Saad H.A., Atta A.A., Alsawat M., Hegab M.S., Altalhi T.A., Refat M.S. (2021). An Environmentally Friendly Method for Removing Hg(II), Pb(II), Cd(II) and Sn(II) Heavy Metals from Wastewater Using Novel Metal–Carbon-Based Composites. Crystals.

[61] El-Shwiniy W.H., El-Desoky S.I., Alrabie A., Abd El-wahaab B. (2022). Spectrophotometric determination of Zr(IV), Hg(II) and U(VI) in solution with their analytical applications: Structural characterization and molecular docking of the solid complexes. Spectrochim. Acta Part A Mol. Biomol. Spectrosc..

